# Potato (*Solanum tuberosum* L.) Leaf Extract Concentration Affects Performance and Oxidative Stress in Green Peach Aphids *(Myzus persicae* (Sulzer)

**DOI:** 10.3390/plants11202757

**Published:** 2022-10-18

**Authors:** Peter Quandahor, Yuping Gou, Chunyan Lin, Changzhong Liu

**Affiliations:** 1 College of Plant Protection, Gansu Agricultural University, No. 1 Yingmen Village, Anning District, Lanzhou 730070, China; 2 CSIR-Savanna Agricultural Research Institute, Tamale P.O. Box 52, Ghana

**Keywords:** potato cultivar, botanical control, detoxifying enzyme, oxidative stress

## Abstract

This study was conducted to determine the aphicidal effect of a leaf extract of the Atlantic potato cultivar on the performance of green peach aphids. Three concentrations of the leaf extract (100, 75, and 50% potato extract), synthetic pesticide (Beta cypermethrin 4.5%), and distilled water (control) treatments were applied in a greenhouse experiment. The results showed that the synthetic pesticide, which was used as a standard check, caused the maximum aphid mortality, followed by the 100% potato leaf extract. Compared with the other botanical treatments, the 100% extract produced low mean rates of survival, aphids’ average daily reproduction, the number of nymphs per plant, and the number of nymphs per adult. This treatment also increased the accumulation of hydrogen Peroxide (H_2_O_2_) and malondialdehyde (MDA), glutathione-s-transferase, mixed-function oxidase, and carboxylesterase content in the green peach aphid. Moreover, the 100% extract also protected the host plants against green peach aphid attacks by demonstrating higher chlorophyll content, net photosynthesis, above-ground fresh weight, and above-ground dry weight of the host plant. This study demonstrates that the highest concentration of potato (Atlantic cultivar) leaf extract (100% extract) could be used as the appropriate dosage for the control of green peach aphids on potatoes, which could greatly reduce the use of synthetic insecticides and promote ecosystem sustainability.

## 1. Introduction

The indiscriminate use of synthetic pesticides results in the development of biotypes, makes minor pests become major pests, produces unfavourable environmental effects, and leads to the development of resistance against a number of insecticides, as well as resulting in ecological inequity, the contamination of food, and the pollution of the environment [1,2]. For instance, due to indiscriminate pesticide use, *Helicoverpa armigera* developed resistance to several pesticide groups [3]. Alternative approaches to insect pest control comprise botanicals that have been shown to be efficient for pest management without posing major challenges. Botanical pesticides are organic or natural insecticides derived from plants of various families and used as plant extracts, essential oils, or both. These natural toxins are a new category of pesticide, which contain molecules that are environmentally and toxicologically safer than several pesticides currently in use [4]. They are less toxic than conventional pesticides and typically affect only the target pests by exhibiting diverse biological actions, subsequently preventing resistance development in the exposed pest populations [5]. Botanicals decompose rapidly in sunlight, moisture, and air due to enzyme detoxification. This demonstrates less persistence and a lower threat to non-target organisms [4]. The use of biopesticides could thus be a feasible way of protecting humans and the environment from the deleterious effects from chemical pesticides [6]. Because organic farming strictly prohibits the use of synthetic chemicals, the use of botanical pest management is recommended, as this promotes natural enemies of pests in the field by increasing species diversity [7]. Thus, organic farming, which promotes the use of botanical pesticides, is regarded as a great alternative that is more environmentally friendly, long-lasting, and can be used to reduce pests in crops [8].

Plants in the Solanaceae family produce secondary metabolites, such as allelochemicals, which are natural protectants against insect pests [9]. Insecticidal constituents are produced during the synthesis of allelochemicals in host plants, which act as deterrents to insect pests [10]. Pure allelochemicals can be used as an alternative to synthetic pesticides for insect pest management [11,12]. Potato (*Solanum tuberosum* L.) plants have been found to produce a variety of secondary metabolites, including glycoalkaloids, which have a plant defense mechanism [13]. The two most common glycoalkaloids found in commercially grown potato varieties are α-chaconine and α-solanine [14]. Because of the possible synergisms between several compounds, crude leaf extracts may have a greater insecticidal effect than individual allelochemicals [15].

Plant allelochemical defense is primarily associated with an increase in reactive oxygen species (ROS) and lipid peroxidation, both of which damage insect cells [16]. In Drosophila melanogaster, increased oxidative stress results in a high mortality rate [16]. To avoid or alleviate cell damage caused by oxidative stress due to insecticide exposure, insect pests stimulate their detoxifying enzymes [17]. Insects are also protected against insecticides by detoxifying enzymes such as glutathione-s-transferase, mixed-function oxidase, and carboxylesterase [18].

Plants respond and adapt to stress conditions through the induction of various morphological and physiological responses [19]. Many physiological factors may be involved in the damage of the photosynthetic apparatus, which decreases leaf chlorophyll content and plant biomass [4], as well as water content [20]. These modifications also have an impact on aphid physiology on host plants [21]. Green peach aphids and other phloem-feeding insect pests remove nutrients and sap from plants, reducing their water potential [22]. Some secondary metabolites of plants act as repellents, antifeedants, and sterilants [5]. This can affect the insects’ physiology, such as water content [23] and reactive oxygen species [24].

Potatoes are one of the world’s most important food crops [25,26]. The green peach aphid, *Myzus persicae* (Sulzer), is a major threat to potatoes due to its ability to adapt to a wide range of plant species [27] and easily develop an anti-insecticide biotype to maintain homeostasis [28]. In addition to being a significant plant pest, green peach aphids also serve as a vector for potyviruses, resulting in significant production losses [29,30]. When *Myzus persicae* feeds on the plant epidermis, it transmits potato virus Y, cucumber mosaic virus [31], and PVY in a non-persistent mode, making management a major concern [32]. Green peach aphids may cause direct and indirect damage, for example they may remove phloem sap directly, and honey dew produced as a nitrogenous waste may indirectly affect photosynthetic and respirational functions by encouraging the development of many sooty moulds. The green peach aphid is considered a serious problem due to its ability to attack a wide range of plant species while impeding crop production. Green peach aphids cause shoot deformation and the abortion of fruits or flowers, distort normal plant growth, and ultimately cause economic damage to crops if not properly controlled [33,34]. Despite being the world leader in potato production, China’s yield per area is lower than the global average due to insect pest damage, including that caused by aphids [26,35]. As a result, a wide variety of synthetic insecticides are constantly used to control aphids in potatoes; however, the associated risks have become a major concern, and synthetic insecticides must be replaced with botanical biopesticides as environmentally benign strategies [36]. In a previous study, we discovered that the drought-sensitive Atlantic genotype exhibited greater tolerance to peach aphids under both drought and well-watered conditions [23]. This cultivar also had high glycoalkaloid content (α-chaconine and α-solanine), which inhibits aphid performance [37]. As a result, we speculate that the Atlantic cultivar leaf extract will inhibit aphid growth and development. This study is based on the hypothesis that the application of potato (Atlantic cultivar) leaf extract will inhibit the performance of the green peach aphid. The objective of this study was to determine the aphicidal effect of potato leaf extract (Atlantic cultivar) on the performance of the green peach aphid.

## 2. Results

### 2.1. The Effect of Potato Leaf Extract on Mean Aphid Survival

**Figure 1:**
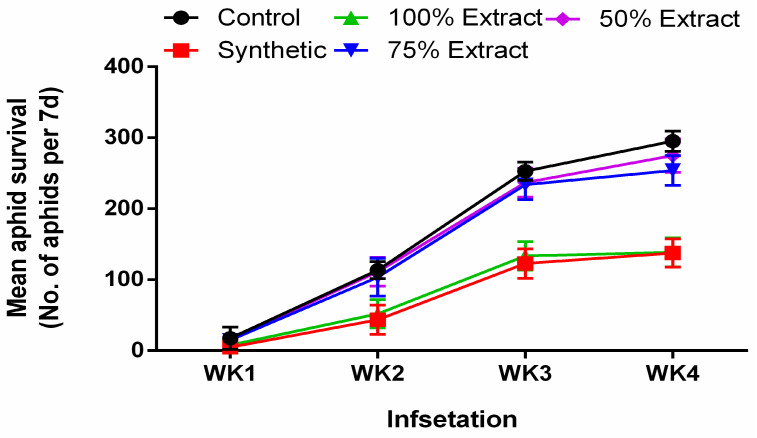
The effect of potato leaf extract on mean aphid survival. Data represent the mean ± standard deviation of three replicates. Note: synthetic pesticide (recommended rate of Beta cypermethrin).

All treatments showed a significant (*p* < 0.01) aphicidal effect regarding mean aphid survival, compared with the control (Figure 1). Generally, the aphid population increased across all treatments over time. However, the treatments did not differ significantly (*p* = 0.06) in terms of the aphid population in the first week. After the first week of treatment, there was a significant (*p* < 0.01) effect on the aphid population during the second week. However, aphid survival under the 50% extract was not significantly (*p* = 0.06) different compared to the control. During the second week, the aphid population was highest in the control group (22.6%) followed by the 50% extract (22.4%), 75% extract (20.2%), and 100% extract (10.3%) groups, and lowest in the synthetic group (8.6%). All of the treatments showed a significant (*p* < 0.01) effect on the aphid population in the third week. However, the aphid population did not differ (*p* > 0.05) between the 50% extract and the 75% extract. In the third week, the aphid population was highest in the control (50.5%), followed by the 50% extract (47.3%), 75% extract (46.7%), and 100% extract (26.7%) groups, and was lowest in the synthetic group (24.5%). Generally, all of the treatments significantly (*p* < 0.01) affected the aphid population in the fourth week. Conversely, the aphid population did not significantly (*p* > 0.05) differ between the 50% extract and 75% extract groups. Comparatively, by the fourth week, the aphid population was highest in the control (distilled water) (56.2), followed by the 50% extract (53.4%), 75% extract (53.3%), and 100% extract (14.0%) groups, and was lowest in the synthetic group (6.9%). Relative to the botanical control treatments, the 100% extract was most lethal to the green peach aphid.

### 2.2. The Effect of Potato Leaf Extract on Aphid Reproductive Performance

**Figure 2:**
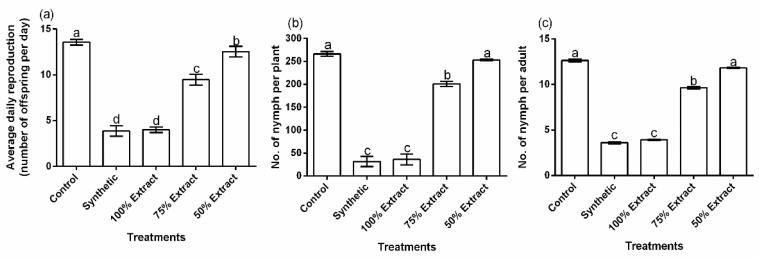
Effect of potato leaf extract on (**a**) average daily reproduction, (**b**) the number of nymphs per plant, and (**c**) the number of nymphs per adult. Data represent the mean ± standard deviation of three replicates. Lowercase letters indicate means that are significantly different according to the LSD test (*p* < 0.05). Note: “differing *Y*-axis scales”, synthetic pesticide (recommended rate of Beta cypermethrin).

All of the treatments showed a significant (*p* < 0.01) effect on aphids’ average daily reproduction, the number of nymphs per plant, and the number of nymphs per adult (Figure 2a–c). However, aphids’ average daily reproduction, the number of nymphs per plant, and the number of nymphs per adult did not significantly (*p* > 0.05) differ between the synthetic pesticide and the 100% extract. Compared with the control, average daily reproduction decreased by 71.4, 70.5, 42.3, and 22.3% under the synthetic pesticide, the 100% extract, the 75% extract, and the 50% extract, respectively (Figure 2a). The number of nymphs also decreased by 81.0, 82.4, 24.5, and 5.0% in the synthetic pesticide, 100% extract, the 75% extract, and the 50% extract, respectively, compared to the control (Figure 2b). The number of nymphs decreased by 70.5, 69.6, 23.7, and 6.3% in the synthetic group, the 100% extract, the 75% extract, and the 50% extract, respectively, compared to the control (Figure 2c). Relative to the botanical control treatments, the 100% extract had the greatest effect on aphid reproductive performance.

### 2.3. The Effect of Potato Extract on Aphid Water Content, the Aphid Tolerance Index, and Relative Water Content

**Figure 3:**
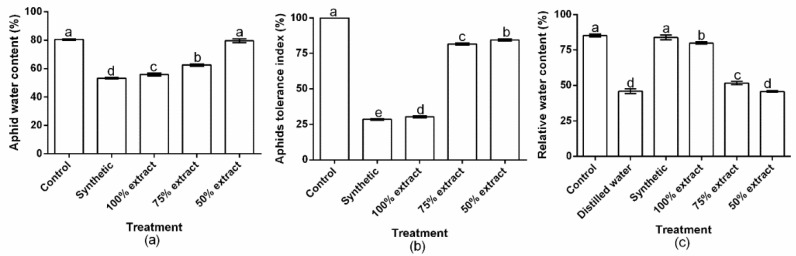
Effect of potato leaf extract on (**a**) aphid water content, (**b**) aphid tolerance index, and (**c**) relative water content. Data represent the mean ± standard deviation of three replicates. Lowercase letters indicate means that are significantly different according to the LSD test (*p* < 0.05). Note: synthetic pesticide (recommended rate of Beta cypermethrin).

Plant leaf and insect water contents are important physiological traits that can indicate the level of stress in plants and insects, respectively (Quandahor 2019; 64). To associate these functional attributes with the host plants and the aphid tolerance exhibited against the various treatments, relative water content, aphid water content, and the aphid tolerance index were analyzed. The results of this study showed significant (*p* < 0.01) treatment effects on aphid water content, the aphid tolerance index, and relative water content across all treatments (Figure 3a–c). Relative aphid water content was 53.2, 55.7, 62.5, and 79.6% in synthetic, 100% extract, 75% extract, and 50% extract groups, respectively (Figure 3a). The highest tolerance index occurred in the 50% extract (84.3%), followed by the 75% extract (81.5%) and the 100% extract (30%), whereas the lowest occurred for the synthetic pesticide (28.4%) (Figure 3b).Generally, the treatments had a significant effect (*p* < 0.01) on relative water content. Conversely, relative water content did not significantly (*p* > 0.05) differ between the synthetic pesticide and the control. Comparatively, the highest relative water content also occurred in the control (84.1) and the synthetic pesticide (83.8%), followed by the 100% extract (79.8), the 75% extract (51.6%), and the 50% extract (45.9%), while the lowest occurred in distilled water (45.8%) (Figure 3c). Relative to the botanical control treatments, the lowest aphid water content and tolerance index and the highest relative water content occurred in the 100% extract. This indicates that the green peach aphid showed more resilience to the other botanical control treatments compared to the 100% extract.

### 2.4. The Effect of Potato Extract on H_2_O_2_ and MDA Content in the Green Peach Aphid

**Figure 4:**
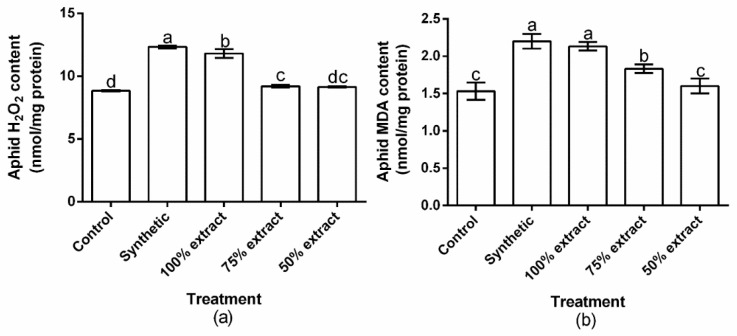
The effect of potato leaf extract on (**a**) aphid hydrogen peroxide content and (**b**) aphid malondialdehyde content. Data represent the mean ± standard deviation of three replicates. Lowercase letters indicate means that are significantly different according to the LSD test (*p* < 0.05). Note: synthetic pesticide (recommended rate of Beta cypermethrin).

The tested treatments showed significant (*p* < 0.02) effects for the accumulation of H_2_O_2_ and MDA in green peach aphids (Figure 4a,b). However, the accumulation of H_2_O_2_ in aphids did not significantly (*p* > 0.05) differ between the 75% extract and the 50% extract. Moreover, the accumulation of MDA in aphids did not significantly (*p* > 0.05) differ between the synthetic pesticide and the 100% extract. Compared to the control, H_2_O_2_ content increased by 36.6, 33.5, 4.1, and 3.3% in the synthetic pesticide, the 100% extract, the 75% extract, and the 50% extract, respectively (Figure 4a). The MDA content also increased by 30.3, 28.1, 16.3, and 4.1% in the synthetic pesticide, 100% extract, 75% extract, and 50% extract, respectively, compared with the control (Figure 4b). Relative to the botanical control treatments, the highest H_2_O_2_ and MDA contents occurred in the 100% extract, whereas the least occurred in the 50% extract.

### 2.5. The Effect of Potato Extract on Green Peach Aphid Detoxifying Enzymes

**Figure 5:**
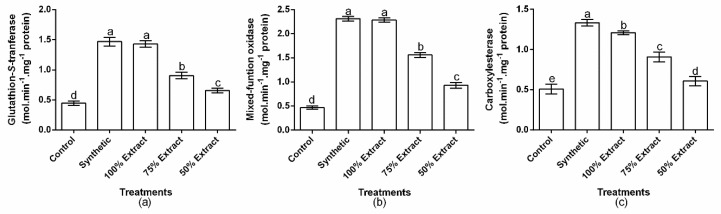
The effect of potato leaf extract on (**a**) glutathaionstransferase, (**b**) mixedfunction oxidase, and (**c**) carboxylesterate. Data represent the mean ± standard deviation of three replicates. Lowercase letters indicate means that are significantly different according to the LSD test (*p* < 0.05). Note: synthetic pesticide (recommended rate of Beta cypermethrin).

The tested treatments showed a significant (*p* < 0.01) effect on the accumulation of glutathione-s-transferase_,_ mixed-function oxidase, and carboxylesterase in green peach aphids (Figure 5a–c). However, the mixed-function oxidase content in aphids did not significantly (*p* > 0.05) differ between the synthetic pesticide and the 100% extract. Compared to the control, glutathione-s-transferase content increased by 65.3, 61.9, 38.1, and 16.1% in the synthetic pesticide, 100% extract, 70% extract, and 50% extract groups, respectively (Figure 5a). The mixed-function oxidase content also increased by 70.3, 68.1, 48.3, and 34.1% in the synthetic pesticide, 100% extract, 75% extract, and 50% extract groups, respectively, compared with the control (Figure 5b). The carboxylesterase content also increased by 61.8, 58.0, 44.0, and 16.4% in the synthetic pesticide, 100% extract, 75% extract, and 50% extract treatments, respectively, compared with the control (Figure 5c). Relative to the botanical control treatments, the highest glutathione-s-transferase, mixed-function oxidase, and carboxylesterase content occurred in the 100% extract, whereas the least occurred in the 50% extract.

### 2.6. The Effect of Potato Leaf Extract on the Physiology of the Host Plant

**Figure 6:**
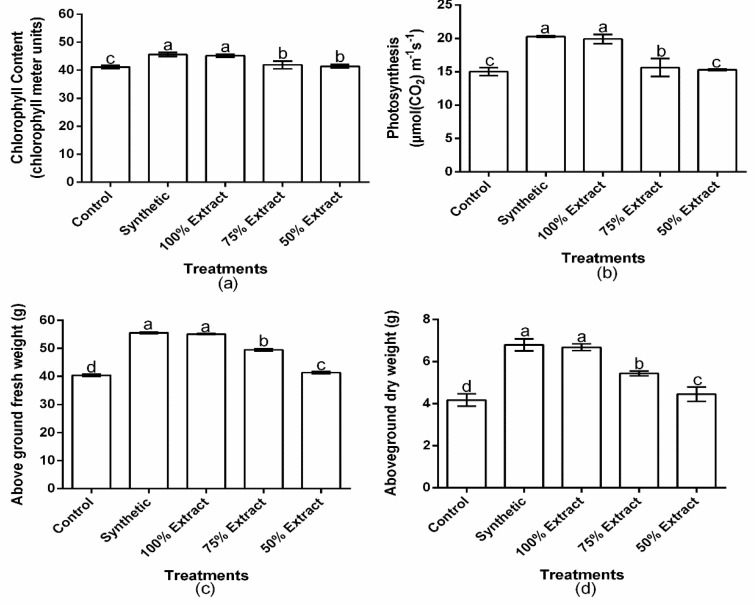
Effect of potato leaf extract on (**a**) chlorophyll content, (**b**) photosynthesis, (**c**) aboveground fresh weight, and (**d**) aboveground dry weight. Data represent the mean ± standard deviation of three replicates. Lowercase letters indicate means that are significantly different according to the LSD test (*p* < 0.05). Note: synthetic pesticide (recommended rate of Beta cypermethrin).

The treatments applied significantly (*p* < 0.01) influenced the chlorophyll content, net photosynthesis, above-ground fresh weight, and above-ground dry weight of the host plant. However, chlorophyll content, net photosynthesis, above-ground fresh weight, and the above-ground dry weight of the host plant did not significantly (*p* > 0.05) differ between the synthetic treatment and the 100% extract (Figure 6a–d). Compared to the control, chlorophyll content decreased by 3.1, 4.1, 11.1, 12.1, and 12.6% in the synthetic, 100% extract, 75% extract, 50% extract, and distilled water treatments, respectively (Figure 6a). Net photosynthesis also decreased by 9.9, 11.5, 30.5, 32.0, and 33.1% in the synthetic, 100% extract, 75% extract, 50% extract, and distilled water treatments, respectively, compared with the control (Figure 6b). The aboveground fresh weight decreased by 0.3, 1.1, 11.2, 25.8, and 27.5% in the synthetic, 100% extract, 75% extract, 50% extract, and distilled water treatments, respectively, compared with the control (Figure 6c). The aboveground dry weight decreased by 0.1, 1.8, 20.1, 34.5, and 38.7% in the synthetic, 100% extract, 75% extract, 50% extract, and distilled water treatments, respectively, compared with the control (Figure 6d). Relative to the botanical control treatments, the highest chlorophyll content, net photosynthesis, aboveground fresh weight, and aboveground dry weight of the host plant occurred in the 100% extract, whereas the least occurred in the 50% extract.

### 2.7. Effect of Potato Extract on H_2_O_2_ and MDA Content in the Host Plant

**Figure 7:**
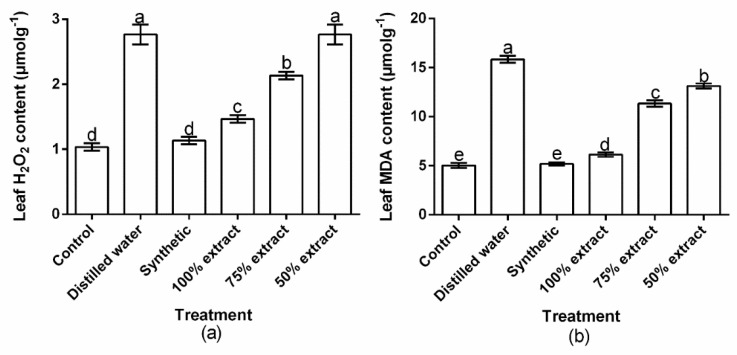
The effect of potato leaf extract on (**a**) leaf hydrogen peroxide (H_2_O_2_) content and (**b**) leaf malondialdehyde (MDA) content. Data represent the mean ± standard deviation of three replicates. Lowercase letters indicate means that are significantly different according to the LSD test (*p* < 0.05). Note: synthetic pesticide (recommended rate of Beta cypermethrin).

The tested treatments showed significant (*p* < 0.01) effect on the accumulation of H_2_O_2_ and MDA in the host plant (Figure 7a,b). However, the accumulation of H_2_O_2_ in plants did not significantly (*p* > 0.05) differ between distilled water and the 50% extract. Moreover, the accumulation of H_2_O_2_ and MDA in plants did not significantly (*p* > 0.05) differ between the synthetic treatment and the control. Compared to the control, the H_2_O_2_ content increased by 8.8, 15.5, 51.5, 62.6, and 62.6% in the synthetic, 100% extract, 75% extract, 50% extract, and distilled water treatments, respectively (Figure 7a). The MDA content increased by 2.5, 10.5, 55.5, 61.6, and 68.2% in the synthetic, 100% extract, 75% extract, 50% extract, and distilled water treatments, respectively, compared with the control (Figure 7b). Relative to the botanical control treatments, the highest H_2_O_2_ and MDA content occurred in the 50% extract, whereas the lowest occurred in the 100% extract.

## 3. Discussion

Botanical insecticides have different effects on different insects depending on the physiological characteristics of the insects and the insecticidal properties of the plant extract [38]. Botanical pesticides prevent insects from feeding by making the treated material unpalatable, causing them to remain on the treated material indefinitely and eventually starve to death [36,38]. In the present study, both the synthetic pesticide and the various concentrations of potato leaf extract reduced the aphid population over time. The 100% potato leaf extract was the most lethal to the green peach aphid, while the 50% potato leaf extract was the least lethal. Despite the common opinion that synthetic pesticides are more effective than botanical pesticides, studies that compared lethal and sublethal toxicities have shown wide variation in results [39,40,41]. Although the synthetic pesticide demonstrated the greatest aphicidal effect on green peach aphids, the 100% potato leaf extract also exhibited similarly significant deleterious effects on green peach aphids. This indicates that botanical pesticides could be as effective as synthetic pesticides, as reported earlier [41]. After four weeks of infestation, the 100% potato leaf extract produced a lower mean rate of survival, which was significantly different from the control population. Secondary metabolites found in *Khaya senegalensis* leaf extracts caused high mortality in Dinoderus porcellus [42]. Adult mortality may be due to contact toxicity or an abrasive effect on the pest cuticle [43], which may also interfere with the insect’s respiratory mechanisms [43]. This suggests that 100% potato leaf extract contain a high concentration of glycoalkaloids, which likely reduced the population of the green peach aphid. When compared to the other levels of botanical pesticides, this level of potato extract also inhibited the reproductive performance of the green peach aphid by reducing the aphid’s average daily reproduction, the number of nymphs per plant, and the number of nymphs per adult. Abdullah et al. [44] obtained a similar result, reporting that 1,8-cineol found in Galangal essential oil had a toxic effect on the reproductive performance of Asian Subterranean Termites, *Coptotermes gestroi*, and *Coptotermes curvignathus*. This confirms that the 100% potato extract contains compounds that inhibit the reproductive potential of the green peach aphid. The other levels of potato leaf extract (75 and 50%) performed poorly when compared to the 100% leaf extract, most likely due to their highly diluted aphicidal constituents.

Plants respond and adapt to stress conditions through the induction of various morphological and physiological responses [19], such as decreases in the relative water content [23]. This modification also has an impact on aphid physiology on host plants [21]. Green peach aphids and other phloem-feeding insect pests remove nutrients and sap from plants, reducing their water potential [22]. The aphid tolerance index, which is used to determine aphid resilience to stress, also showed that the 100% potato leaf extract was more effective against the green peach aphid. Moreover, host plants treated with the 100% potato leaf extract had a higher relative water content, and aphids on these plants had a lower water content. Some secondary metabolites of plants act as repellents, antifeedants, and sterilants [5]. This can affect the insect’s physiological characteristics, such as water content [23]. This suggest that the 100% potato leaf extract possibly contained sufficient amounts of secondary metabolites to repel the aphids from feeding on the host plant phloem. The extreme water loss of the aphids on these plants also indicate how the aphids were starved regardless the water content; this could be due to the level of repellent in the extract.

It is reported that the plant allelochemical defense mechanism is mostly related to the elevation of ROS and lipid peroxidation, which damage insect cells [16]. Botanical pesticides increased oxidative stress in two lepidopteran species [45,46,47] and R. *lophanthae* [48]. This was particularly true for mineral oil, which also resulted in the highest levels of mortality among the pesticides evaluated for oxidative effects [48]. In the present study, the highest accumulation of H_2_O_2_ and MDA content occurred in the 100% potato leaf extract, whereas the least occurred on the 50% potato leaf extract, relative to the botanical pesticides. This suggests that the 100% potato leaf extract treatment had a greater aphicidal effect on green peach aphids, which possibly led to the high mortality rate by causing high levels of oxidative damage to the aphids, compared with the other botanical pesticides. A similar result was reported in *Drosophila melanogaster*, where high levels of oxidative damage increased the mortality rate [16]. Herbivore insects exhibit a complex detoxifying enzyme to reduce the flux of oxidative radicals generated by botanical pesticides [17,49]. This has been reported in many insect species [10,17]. In the present study, all of the tested treatments showed some level of effect on the accumulation of glutathione-s-transferase, mixed-function oxidase, and carboxylesterase in green peach aphids, except for the control. The highest glutathione-s-transferase, mixed-function oxidase, and carboxylesterase content levels occurred in the 100% potato leaf extract, compared with the other botanical pesticides. The greater elevation of the detoxifying enzymes observed in green peach aphids reared on 100% potato leaf extract shows that the aphids possibly developed defense mechanisms, to some extent. However, this was not sufficient to defend them from the lethal effects of this treatment. This indicates that the detoxifying enzymes could not reduce the oxidative radicals generated by the 100% potato leaf extract.

Host plants adapt to stress conditions through the orientation of various physiological and morphological changes [50]. Stress conditions can affect the photosynthetic apparatus and reduce leaf chlorophyll content [51], as well as the aboveground biomass [52]. In the current study, all treatments had some level of deleterious effect on chlorophyll content, net photosynthesis, aboveground fresh weight, and the dry weight of the host plant. Relative to the botanical pesticides, the greatest chlorophyll content, net photosynthesis, aboveground fresh weight, and aboveground dry weight of the host plant occurred in the 100% potato leaf extract. This suggest that the 100% potato leaf extract possibly repelled the aphids from feeding on the host plant phloem, thereby protecting the host plant’s physiology and morphology from aphid stress. This was also confirmed by the low accumulation of H_2_O_2_ and MDA content, which are reported as an early biochemical signal of oxidative stress responses in plants under stress conditions [53].

## 4. Materials and Methods

### 4.1. Growth Conditions and Planting Materials

The experiment was conducted in a greenhouse (day temperature 25–35 °C, night temperature 18–22 °C, daytime relative humidity 45–55%, light intensity 15,000–18,000 lux) at Gansu Agricultural University, Lanzhou, China. Miniature tubers of Qingshu 9 (host plant) and Atlantic (leaf extract) potato cultivars were obtained from the Gansu Haofeng Seed Company Limited of Lanzhou, China for the experiment. The tubers were sown in pots (20 cm diameter, 15 cm high) filled with 2 kg of loamy soil.

### 4.2. Aphid Culture

Adult *Myzus persicae* were collected from potato plants at the experimental farm of Gansu Agricultural University in Lanzhou, China. These aphids were reared on potato plants in ventilated glass cages. The culture was maintained in an a controlled environment at 19 ± 1 °C under a 16:8 h light:dark photo cycle. The culture was preserved for six months before being used for the experiment.

### 4.3. Experimental Design and Treatments

The experiment had a completely randomized design, with six application treatments replicated three times. The experiment involved three levels of botanical control treatment (100, 75, and 50% potato extract), a synthetic chemical pesticide (recommended rate of Beta cypermethrin, 4.5% as standard check), distilled water (control for aphids indexes), and aphid-free plants (control for host plant indexes). This experiment utilized two potato cultivars of medium relative maturity (40 d). A total of 144 pots were used for the experiment and each experimental unit consisted of eight pots with one plant per pot. In each experimental unit, eight plants were sampled for data collection, giving eight subsamples in each of the three replications for each treatment. The plants were watered adequately to maintain the soil moisture at field capacity. Weeds that occasionally emerged were controlled regularly by hand-picking to keep pots free of weeds.

### 4.4. Preparation and Application of the Potato Leaf Extract

The extract was prepared according to the method described by Marciniak et al. [54], with slight modifications. In brief, fresh leaves of potato plants (Atlantic cultivar) were harvested 40 d after planting and immediately stored at −20 °C. The leaves were ground using a laboratory mill. The suspension was centrifuged at 3828 RCF, 4 °C, for 30 min. The liquid extract was filtered through single-use 0.22 μm nylon filters (Whatman, Maidstone, UK). The botanical treatments (100, 75, and 50% potato extract), the synthetic pesticide (recommended rate of Beta cypermethrin, 4.5%) treatment, and the distilled water treatment were sprayed using an atomized hand sprayer. Treatment application was conducted in 7-d intervals for a period of four weeks.

### 4.5. Determination of Aphid Performance

Forty-day-old potato plants were infested with aphid nymphs and monitored for 28 d. Eight shoots of each treatment were infested with 20 nymph aphids (*n* = 20 for each treatment). Mean aphid survival was measured by counting the number of aphids that remained alive on each plant during the period of 28 d after infestation. The rate of survival was recorded after every seven days for four weeks. Average daily reproduction was determined as the number of nymphs divided by the number of days for the period of infestation. The number of nymphs per plant was assessed by counting the number of nymphs produced on each plant up to the 28th day after infestation. The number of nymphs produced on a plant was divided by the number of adults per plant at 28 d after infestation to assess the number of nymphs per adult.

### 4.6. Aphid Water Content (AWC) and the Aphid Tolerance Index (ATI)

To determine aphid tolerance against the various treatments, aphid water content and the aphid tolerance index were analyzed. Aphid water content was determined as described by Guo et al. [55]. Adult aphids from each plant were collected 20 d after infestation and weighed immediately, dried at 60 °C for 24 h, and then weighed again. Water content was determined by deducting the dry weight from the fresh weight of each aphid. Aphid water content was calculated as:

(1) $$\mathrm{AWC}=\frac{\mathrm{FW}-\mathrm{DW}}{\mathrm{DW}}\times 100$$

The aphid tolerance index (ATI), which indicates the resilience of the aphids to pesticides, was calculated according to Wilkins [56] as follows:

(2) $$\mathrm{ATI}=\frac{\mathrm{Aphids}\;\mathrm{reared}\;\mathrm{on}\;\mathrm{treated}\;\mathrm{plants}}{\mathrm{Aphids}\;\mathrm{reared}\;\mathrm{on}\;\mathrm{control}\;\mathrm{plants}}\times 100$$

### 4.7. Determination of Hydrogen Peroxide (H_2_O_2_) and Malondialdehyde (MDA) Content in Aphids

The frozen aphid samples were homogenized in a cold mortar with a pestle in a 0.05 M phosphate-buffered solution, pH 7.8, containing 0.1 mM ethylenediamine tetraacetic acid and 1% polyvinylpyrrolidone. The crude homogenates were centrifuged at 10,000× *g* for 15 min at 4 °C. The supernatant was collected and used for the concentration determination by spectrophotometry (Shimadzu UV-2450, Arlington, MA, USA). The H_2_O_2_ and MDA contents were determined following the methods of Zhu et al. [57].

### 4.8. Determination of Aphids’ Detoxifying Enzymes

The frozen aphids (10 adults per treatment) were homogenized in 1 mL of 0.1 M phosphate buffer (pH 7.0) at 4 °C. The crude homogenate was centrifuged at 13,000× *g* for 15 min at 4 °C. The supernatants were collected and used to examine the activities of the three major detoxifying enzymes: carboxylesterase, glutathione-s-transferase, and mixed-function oxidase. Each analysis was replicated three times. The enzyme activity of carboxylesterase was determined as described by Cui et al. [58], and total activity was quantified using a Beckman DU-800 spectrophotometer (Becton-Dickinson, Fullerton, CA, USA). Glutathione-s-transferase enzyme activity was determined as described by Sun et al. [59] and mixed-function oxidase enzyme activity was determined following the methods described by Sintim et al. [60]. Total protein concentrations in the above-mentioned supernatants were determined using Bradford’s method [61].

### 4.9. Leaf Relative Water Content (RWC)

To determine the host plants’ levels of stress against aphid infestation, leaf water content was analyzed. Leaf relative water content was determined according to the method of Barrs and Weatherley [62]. Four leaves (the youngest fully expanded leaves) were harvested from eight shoots of each treatment. The fresh weight (FW) was measured immediately after harvest. The leaves were submerged in distilled water for 6 h. The leaves were then removed, and the adhering water was blotted with tissue paper before weighing to obtain turgor weight (TW). The leaves were oven dried to a constant weight at 70 °C in an oven for 24 h to measure the dry weight (DW). The relative water content (RWC) was calculated as follows:

(3) $$\mathrm{RWC}=\frac{\mathrm{FW}-\mathrm{DW}}{\mathrm{TW}-\mathrm{DW}}\times 100\%$$

### 4.10. Chlorophyll Content and Net Photosynthesis

Eight shoots of each treatment were selected to measure leaf chlorophyll content and net photosynthesis. The measurement was taken on the fifth to tenth terminal mature leaves from the base of the shoot 20 d after infestation. A portable chlorophyll meter (CCM-200, Opti-Sciences, Tyngsboro, MA, USA) was used to measure leaf chlorophyll content. Light-saturated net photosynthesis was also measured with a portable infrared gas analyzer (LI-6200, LI-COR, Lincoln, NE, USA).

### 4.11. Determination of the Aboveground Biomass

The aboveground biomass was determined as described previously by Bañón et al. [63]. The aboveground fresh shoot weight was determined immediately after harvest. The harvested fresh shoots were oven dried to a constant weight at 80 °C for 72 h to determine the aboveground dry weight per plant.

### 4.12. Determination of H_2_O_2_ Content in Leaves

Hydrogen peroxide levels in leaf samples were determined as described previously by Mostofa and Fujita [64]. Leaf samples were homogenized in an ice bath with 5 mL 0.1% (*w/v*) trichloroacetic acid (TCA). The homogenate was centrifuged at 12,000× *g* for 20 min. The supernatant (0.5 mL) was mixed with 0.5 mL 10 mM potassium phosphate (K_3_PO_4_) buffer (pH = 7.0) and 1mL 1 MKI. The absorption of the supernatant was measured at 390 nm, and the content of H_2_O_2_ was calculated using the H_2_O_2_ reference standard curve.

### 4.13. Determination of MDA Content in Leaves

Malondialdehyde content in leaf samples was determined according to the method described by Liu et al. [65]. In brief, leaf samples were homogenized in 5% (*w/v*) TCA. The homogenate was centrifuged at 10,000× *g* for 5 min. The supernatant (0.5 mL) was mixed with 1 mL of 0.5% (*w/v*) TBA in 20% TCA. The supernatant was used for the MDA assay.

### 4.14. Statistical Analysis

Statistical analysis was performed using SPSS statistics software (version 19.0, SPSS, Chicago, IL, USA). Treatment means were separated using Duncan’s multiple range test *(p <* 0.05). The results are presented as means ± standard deviation.

## 5. Conclusions

The results show that the synthetic pesticide, which was used as a standard check, caused the maximum aphid mortality, followed by the 100% potato leaf extract. However, the 100% potato leaf extract also exhibited similarly significant deleterious effects on green peach aphids, compared with the other levels of botanical control. This botanical treatment also had a significant effect on the reproductive potential of green peach aphids. We therefore conclude that the lowest dilution of potato (Atlantic cultivar) leaf extract (100% extract) is the appropriate dosage to be used for green peach aphid control. This can greatly reduce the use of synthetic insecticides, which are continuously used and endanger the health of farm operators, animals, and food consumers.

## Data Availability

Not applicable.
